# Mast Cells and MCPT4 Chymase Promote Renal Impairment after Partial Ureteral Obstruction

**DOI:** 10.3389/fimmu.2017.00450

**Published:** 2017-05-04

**Authors:** Maguelonne Pons, Liza Ali, Walid Beghdadi, Luca Danelli, Marianne Alison, Lydia Celia Madjène, Jessica Calvo, Julien Claver, Shamila Vibhushan, Magnus Åbrink, Gunnar Pejler, Marie-Laurence Poli-Mérol, Michel Peuchmaur, Alaa El Ghoneimi, Ulrich Blank

**Affiliations:** ^1^INSERM UMRS 1149, Paris, France; ^2^CNRS ERL8252, Paris, France; ^3^Université Paris Diderot, Sorbonne Paris Cité, Laboratoire d’excellence INFLAMEX, Paris, France; ^4^Department of Pediatric Surgery and Urology, Hôpital Robert Debré, APHP, Université Paris Diderot, Sorbonne Paris Cité, Paris, France; ^5^Department of Pediatric Radiology, Hôpital Robert Debré, APHP, Université Paris Diderot, Sorbonne Paris Cité, Paris, France; ^6^Department of Pathology, Hôpital Robert Debré, APHP, Université Paris Diderot, Sorbonne Paris Cité, Paris, France; ^7^Section of Immunology, Department of Biomedical Sciences and Veterinary Public Health, Swedish University of Agricultural Sciences, Uppsala, Sweden; ^8^Uppsala University, Department of Medical Biochemistry and Microbiology, Uppsala, Sweden; ^9^Swedish University of Agricultural Sciences, Department of Anatomy, Physiology and Biochemistry, Uppsala, Sweden; ^10^Pediatric Surgery Unit, American Memorial Hospital, Université Reims Champagne Ardennes, Reims, France

**Keywords:** mast cells, chymase, hydronephrosis, fibrosis, inflammation

## Abstract

Obstructive nephropathy constitutes a major cause of pediatric renal progressive disease. The mechanisms leading to disease progression are still poorly understood. Kidney fibrotic lesions are reproduced using a model of partial unilateral ureteral obstruction (pUUO) in newborn mice. Based on data showing significant mast cell (MC) infiltration in patients, we investigated the role of MC and murine MCPT4, a MC-released chymase, in pUUO using MC- (W^sh/sh^), MCPT4-deficient (*Mcpt4*^−/−^), and wild-type (WT) mice. Measurement of kidney length and volume by magnetic resonance imaging (MRI) as well as postmortem kidney weight revealed hypotrophy of operated right kidneys (RKs) and compensatory hypertrophy of left kidneys. Differences between kidneys were major for WT, minimal for W^sh/sh^, and intermediate for *Mcpt4*^−/−^ mice. Fibrosis development was focal and increased only in WT-obstructed kidneys. No differences were noticed for local inflammatory responses, but serum CCL2 was significantly higher in WT versus *Mcpt4*^−/−^ and W^sh/sh^ mice. Alpha-smooth muscle actin (αSMA) expression, a marker of epithelial–mesenchymal transition (EMT), was high in WT, minimal for W^sh/sh^, and intermediate for *Mcpt4*^−/−^ RK. Supernatants of activated MC induced αSMA in co-culture experiments with proximal tubular epithelial cells. Our results support a role of MC in EMT and parenchyma lesions after pUUO involving, at least partly, MCPT4 chymase. They confirm the importance of morphologic impairment evaluation by MRI in pUUO.

## Introduction

Obstructive uropathies represent a sizable proportion (~50%) of antenatal renal diseases with ureteropelvic junction (UPJ) obstruction being the first congenital etiology (1, 2). In some cases, the pelvic dilatation may regress spontaneously with age but the others will require surgery. Indication for surgery largely depends on imaging criteria requiring a few months follow-up of patients. Renal function and kidney morphology data are evaluated by renal ultrasound, scintigraphy, and more recently, uro-magnetic resonance imaging (MRI) (3, 4). These examinations are non-invasive, but poorly predict the optimal timing for surgery and renal recovery after surgical repair. A better understanding of the pathophysiology combined with biochemical and imaging investigations is necessary to delineate new biomarkers, evaluate the impact of these lesions in the long term, and improve patient care.

Ureteropelvic junction obstruction decreases urine flow down the ureter with a concomitant increase of fluid pressure inside the kidney. The urinary stasis induces chronic inflammation and progressive destruction of renal parenchyma. The inflammatory phase with cell infiltration and production of inflammatory mediators promotes the activation of myofibroblasts with accumulation of extracellular matrix (ECM) proteins such as alpha-smooth muscle actin (αSMA), fibronectin, and collagen, a process called epithelial–mesenchymal transition (EMT). EMT is followed by development of glomerular sclerosis, tubular atrophy, and tubulointerstitial fibrosis with the final consequences of end-stage organ failure (5, 6). However, UPJ obstruction is a heterogeneous disease with variable consequences, likely depending on the degree of obstruction and parenchyma alterations and may also change with the course of the disease (7).

Mast cells (MCs) are tissue-localized innate immune cells, which have emerged as prime effectors of inflammatory responses and tissue repair in many diseases including kidney diseases (8–11). In human nephropathies, MC infiltration is a prominent feature (12–14). MCs contain, in their cytoplasmic granules, numerous mediators some of which are MC-specific including several proteases such as tryptases, carboxypeptidase A, and chymases. Tryptase was shown to induce fibroblast proliferation and collagen synthesis (15), while chymase degrades different ECM proteins either directly or indirectly through the activation of pro-matrix metalloproteinases (16, 17). Based on studies with MC-deficient animal models, conflicting reports exist about the role of MC in the pathogenesis of kidney diseases. While some studies have revealed a protective or anti-fibrotic role of MC or MC-derived mediators (14, 18, 19), others reported a deleterious effect (20, 21) or pro-fibrotic actions relating to their potential to produce pro-fibrotic mediators and cytokines (22). This suggested that the pathophysiological context of MC action is important and needs to be further explored.

To study the pathophysiology of UPJ obstruction, which generally involves partial obstruction, models such as partial unilateral ureteral obstruction (pUUO) have been developed (23) in neonatal rats (24) and later in neonatal mice (25, 26). As rodent and murine nephrogenesis is incomplete at birth and finalized during the first week of life, this model is close to human congenital uropathies. Murine models also allow the use of transgenic animals more readily available than in the rat (27, 28). Recently, we have been able to generate renal lesions after partial ureteral obstruction in newborn mice at 3 days of life (29). Long-term results confirmed that pUUO in newborn mice produces fibrosis of renal parenchyma (30).

In order to study the involvement of MC and MC mediators, we decided to apply the pUUO experimental model to MC-deficient mice (*kit*^Wsh/Wsh^ mice), and murine MC protease 4-deficient mice (*Mcpt4^−/−^* mice). Among the murine chymases, MCPT4 is most likely the functional counterpart of the unique human chymase because it has similar substrate specificity (17, 21), tissue distribution, proteoglycan-binding properties, and ability to convert angiotensin (Ang) I into Ang II (31, 32). Our results support that MC can significantly contribute to the development of UPJ pathology. This action is at least partially mediated by released MCPT4 chymase.

## Materials and Methods

### Mouse Strains

Male C57BL/6J mice were bred at the Bichat Medical School’s breeding facility. MC-deficient (*kit*^Wsh/Wsh^) in the C57/BL/6J background, named thereafter W^sh/sh^, have been obtained from Juan Rivera (NIH) and are bred at Bichat Medical School’s animal facility. Mice deficient in MCPT4 chymase in the C57/BL/6J maintained in Bichat Medical School’s breeding facility have been described previously (21).

### Patient Samples

Histological kidney specimens of 21 pediatric patients (12 males and 9 females, age 2–168 months) with severe obstructive uropathy of diverse origin who had undergone kidney resection were assayed for the inflammatory infiltrate and fibrosis in retrieved kidney specimens as part of a routine procedure to evaluate kidney damage.

### Animal Surgery

Newborn mice at day 3 of life were placed on a warming pad under a microsurgical microscope. Under anesthesia by isoflurane and oxygen, a right longitudinal flank incision was made to expose the right ureter before embedding it in the psoas muscle by two points, 11-0 nylon sutures, according to the technique described by Ulm and Miller (33). After closure of the incision (9-0 nylon sutures), the mice were allowed to recover on the warming pad with oxygen before returning them to their mother (29). Due to the surgery procedure, there was an important postoperative mortality [wild-type (WT) 82%; W^sh/sh^ 81%; and *Mcpt4^−/−^* 70%] most of which was due through maternal cannibalism. Postoperative deaths were excluded from the study.

### MRI Studies

Twenty-four hours before euthanization, imaging sequences were performed with a 7-T MRI, 12 cm diameter, horizontal bore magnet (Pharmascan, Bruker-Biospin SA, Germany). All MRI sequences were respiratory triggered in order to limit artifact induced by respiratory movement using anesthesia by isoflurane and oxygen (1.5% isoflurane in 1 l/min air/O_2_) administered through a nose cone. Mice body temperature was maintained at 36–37°C with a warm water circulator. The mouse was in the prone position inside a quadrature bird cage coil (Bruker). Morphological analysis of kidneys was performed using a coronal T2-weighted 2D RARE sequence (anatomic acquisition) with the following parameters: TR/TE: 1,500/11 ms; field of view: 40 mm × 40 mm; acquisition matrix: 128^2^; 3 coronal slices; slice thickness: 1 mm; no gap between slices, RARE factor: 8, 2 signal averages, fat suppression. The acquisition was synchronized with respiration using balanced acquisitions over several respiratory periods with an effective TR of about 2.5 s. The acquisition time was around 5 min, depending on breath rate for each mouse. This sequence was optimized to compare the operated kidney with the non-operated kidney. On the coronal plan, the maximum length kidney was measured from the superior to the inferior parenchyma pole. Coronal T2-weighted images were made with a slice thickness of 1 mm without gaps. The kidney parenchyma volume was calculated using ImageJ (W. Rasband, NIH, USA) and the additional Magnetic Resonance Urography plugin, by adding every parenchyma area (contours were manually drawn) multiplied by slice thickness. The number of pixels was determined in each slice through the parenchyma excluding cavities (2D surface). The kidney volume (*V*_t_) was calculated as follows: *V*_t_ = (*A*_1_ + *A*_2_ + … + *A_n_*) × PS × ST^h^ where *A_n_* is the number of pixel of the parenchyma in the slice *n*, PS is the pixel area, and ST^h^ is the slice thickness. The studies were conducted following the recommendations of the European Convention for the protection of Vertebrates Animals used for Experimentation.

### Methylene Blue (Methylthioniniumchloride) Test and Kidney Weight Determination

At day 75, before euthanization, we ensured ureteric permeability on anesthetized mice (100 mg/kg ketamine and 20 mg/kg xylazine, i.p.), by injecting Methylene blue as described (30). Mice with a complete ureteral obstruction were excluded from the study. After vascular perfusion by heart puncture (20 ml PBS), kidneys were removed by blunt dissection and weighed without the upper tract using the same laboratory balance.

### Sample Preparation and Histological/Immunohistochemical Analysis of Animals and UPJ Patients Kidneys

The inferior part of kidney (~25%) was cut and used for analysis of αSMA expression. The remainder was cut into two longitudinal parts. One half was fixed in 4% formalin and embedded in paraffin. Tissue sections (4 µm) were stained with hematoxylin-eosin-safran, Masson’s trichrome, and Sirius Red. Images were observed with a Leica microscope (Leica Microsystems, Rueil-Malmaisons, France) coupled to a MD2000 camera (Leica Microsystems) using a 10×, 20×, 40× auxiliary lens and a direct X1C-mount. Objective magnifications are as indicated. Renal fibrosis, interstitial infiltration, and the number of glomeruli in vertical cortex range were scored. All histological quantifications were performed by an experienced pathologist under magnification 10× (fibrosis) or 20× (infiltration) in a blinded fashion according to standard scores: 0 absence of lesions, 1 when <25% of the slide area, 2 when >25 and <50%, and 3 when >50%. Photographs of Sirius Red stained sections to illustrate renal fibrosis and glomerular ranks were made with an image segmentation software after converting the glass slides into digital slides (ImageScope, Aperio, Vista, CA, USA). MC staining with toluidine blue was performed as described (34). Immuno-histochemical staining was performed according to standard procedures using 4 µm formalin fixed sections. T cell infiltrate in mouse kidney sections was evaluated using a rabbit anti-CD3 (Dako). Macrophage infiltrate in mouse kidney sections was checked using rat anti-F4/80 (clone A3-1) and anti-CD11b (clone M1/70) antibody (BD Biosciences). MC and T cell infiltration in samples from UPJ patients was evaluated using mouse anti-chymase, mouse anti-tryptase, and mouse anti-c-kit antibodies (all AbDSerotec) for MC and a mouse anti-CD3 antibody (clone UCHT-1; DAKO A/S) for T cells as well as Fab’2 anti-mouse-HRP secondary antibodies (Jackson Immuno research). For inflammatory infiltration, the (CD3^+^) Banff score was used to identify three groups of severity of kidney disease with low, intermediate, and high inflammatory cell infiltration (Banff scores 1, 2, and 3). The Banff score (35), which classically is used to measure the degree of rejection in kidney allografts by determining among other criteria the mononuclear T cell infiltrate, was adapted here to score the degree of inflammation in UPJ kidneys. A minimum of three high power fields (hpf) were analyzed per patient. Fibrosis scores were determined using Masson’s trichrome stained sections.

### Immunoblotting

Lysates from left and right kidney tissues were prepared in 50 mmol/l Tris–HCl (pH 8.8) containing 1% sodium dodecyl sulfate and 5% glycerol. A total of 20 µg of kidney lysates were migrated on a 10% sodium dodecyl sulfate-polyacrylamide gel electrophoresis (SDS-PAGE) followed by transfer onto nitrocellulose membrane (Schleicher and Schuell, Dassel, Germany). Membranes were blocked with 4% bovine serum albumin for 1 h followed by incubation for 1 h at room temperature (RT) with the primary antibodies: a purified mouse anti-αSMA-1 (clone 1A4, Thermo Fisher Scientific, France) and anti-αtubulin (Sigma). After several washes, blots were incubated with goat anti-mouse IgG HRP (1/5,000) (Jackson Immunoresearch, Newmarket, UK) for 45 min and were developed by enhanced chemiluminescence; GE, Paris, France. Quantitative analysis of blots was performed by densitometry using NIH ImageJ software. The ratio between αSMA and α tubulin (loading control) was determined and compared to a kidney lysate from a sham-operated WT mouse always run in parallel and arbitrarily set to 1.

### Determination of CCL2 Concentration in Blood Samples

CCL2 concentrations in serum collected at the day of euthanization as well as TGFβ and IL6 concentrations in IgE-sensitized MC stimulated with specific antigen (DNP-HSA at 30 ng/ml) were quantified using commercial ELISA according to the manufacturer’s instructions (Duoset cytokine Elisa Kits, R&D System, Lille, France).

### Cell Culture of Mouse Proximal Tubule Cell (MPTC) and Bone Marrow-Derived MCs (BMMCs) and Production of Supernatants for Coculture Assays

Mouse proximal tubule cells were isolated from 3- to 4-week-old C57Bl/6 mice as described (36). Briefly, kidneys were removed aseptically from anesthetized mice. The cortex was separated from the medulla and incubated for 30 min in a 1 mg/ml collagenase solution. Homogeneous populations of nephron segments were separated on a Percoll gradient (Percoll 42%; centrifugation 17,000 rpm for 30 min at 4°C). The F_4_ layer, composed almost exclusively of proximal tubules, was seeded on glass slides (76 mm × 26 mm) in culture medium [Ham’s F-12/Dulbecco’s modified Eagle’s medium (1:1, vol/vol), 25 mM HEPES, 21.5 mM HCO_3_, 1 mM Na pyruvate, 10 ml/l of a 100 × non-essential amino acid mixture, 4 mM l-glutamine, 50 U/ml penicillin, 50 µg/ml streptomycin, and 50 nM Na selenite] supplemented with 5 µg/ml insulin, 35 µg/ml transferrin, 5 nM triiodothyronine, 0.5 µM retinoic acid, 25 ng/ml prostaglandin E_1_, and 50 nM dexamethasone. Fetal calf serum (1%) was present in the medium for the first 48 h. To analyze induction of alpha-SMA, expression cultures were used at day 7. BMMCs were obtained by isolating bone marrow precursors from femurs and tibias of WT mice and grown as described in medium containing recombinant murine IL-3 and stem cell factor at 10 ng/ml (Peprotech, Paris, France) for 5 weeks to obtain differentiated BMMCs (34). To test the capacity of supernatants for induction of αSMA expression in MPTC in co-culture assays, BMMCs (1 × 10^6^) were sensitized with 1 µg/ml IgE anti-DNP (37) for 3 h in MPTC culture medium and were then either left unstimulated or were stimulated with 30 ng DNP-HSA. Supernatants were collected at indicated time points and added to MPTC cultures for 72 h. TGFβ (Peprotech) at 5 ng/ml was added to MPTC cultures as a positive control.

### Immunofluorescence Analysis of αSMA Expression by MPTC

After co-culture with supernatants from BMMC, MPTC cells (about 1 × 10^5^) were added to 8-well Lab-Tek chamber slides and allowed to adhere before fixing them for 20 min on ice in 10 mM PIPES pH6.8, 150 mMNaCl, 5 mM EGTA, 5 mM MgCl_2_, 5 mM glucose containing 4% paraformaldehyde (IF buffer). After permeabilization in IF buffer containing 0.025% saponin for 20 min at RT, followed by blocking in IF buffer containing 0.012% saponin and 7% horse serum (Invitrogen) for 30 min at RT. Staining with primary mouse anti-αSMA antibody was performed in IF buffer containing 0.012% saponin and 5% horse serum for 2 h at RT followed by incubation with secondary goat anti-mouse FITC Ab for 60 min at RT. Cells were mounted in Prolong-Gold anti-fading reagent (Molecular Probes) and were analyzed under an immunofluorescence microscope. Quantitative analysis of fluorescence was performed by densitometry using NIH ImageJ software.

### Statistical Analysis

Statistical analysis was performed using GraphPad Prism software (release 5.00 windows, GraphPad Software, San Diego, CA, USA). Statistical significance between the three mice strains (WT, W^sh/sh^, *Mcpt4^−/−^*) was examined by Kruskal–Wallis test. To compare two delta-values of the three strains mice (WT, W^sh/sh^, *Mcpt4^−/−^*), a Mann–Whitney test was used. For each mouse strain, comparison between RT and RL was examined by Wilcoxon test. To compare two RT or two RL coming from two different mice strains, a Mann–Whitney test was used. The Spearman correlation test was used to evaluate correlation between volume and weight kidneys for each mouse strain. *p*-Values of 0.05 were considered significant.

## Results

### MCs and Chymase Are Present in “Pediatric” Patients with UPJ Obstruction

**Figure 1:**
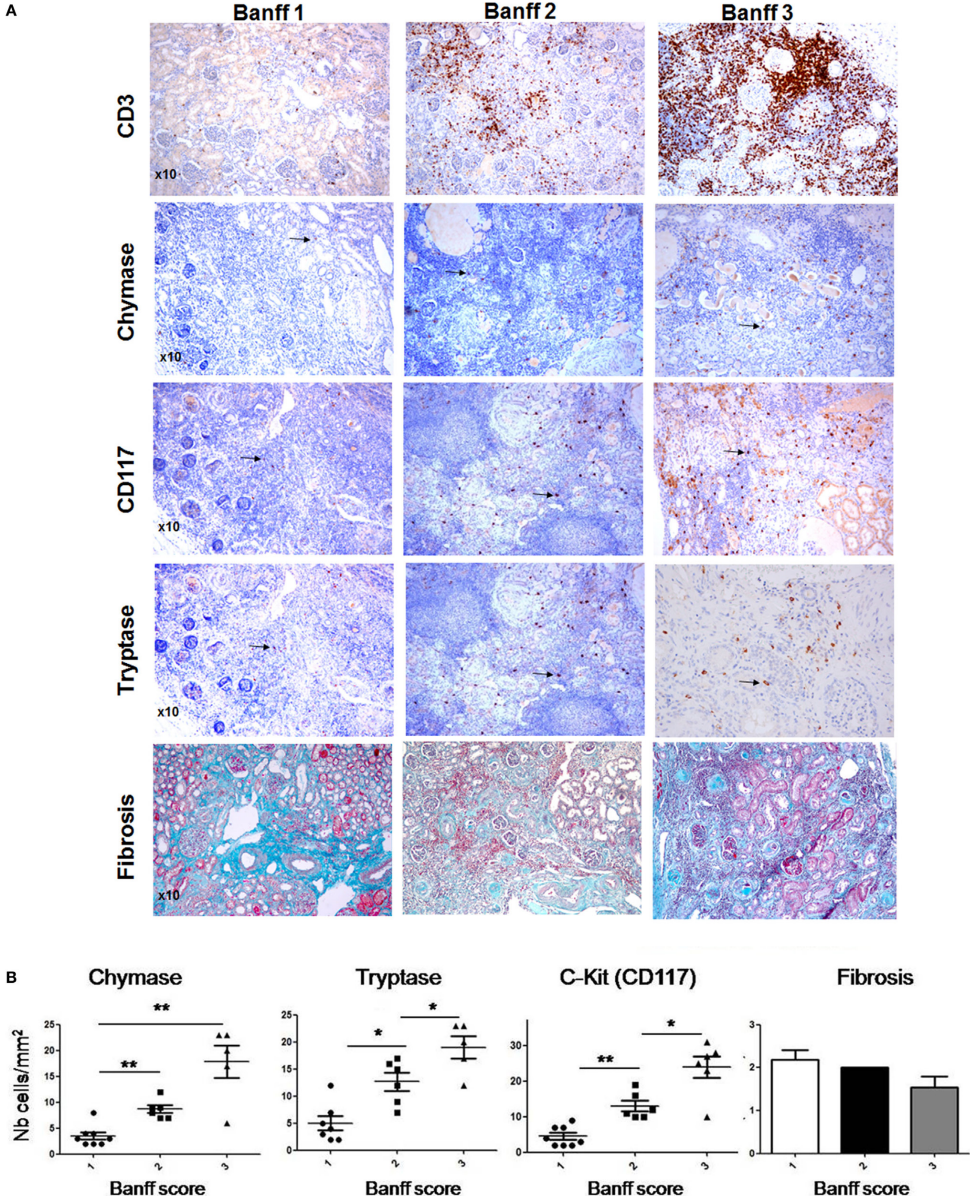
**Evaluation of mast cell (MC) numbers and fibrosis scores in hydronephrosis patients**. **(A)** (Upper panel) Three representative hydronephrosis patients with different degree of the inflammatory CD3^+^ cell infiltrate, showing respectively low, intermediate, and high inflammatory cell infiltrate (Banff scores 1, 2, and 3). (Middle panels) Immunohistochemical analysis of MCs using an anti-chymase, anti-CD117, and anti-tryptase antibodies in sections with Banff scores 1, 2, and 3. Arrows point to MCs. Three representative Masson’s trichrome stained sections to evaluate fibrosis in sections with Banff scores 1, 2, and 3. **(B)** Quantitative analysis of MC numbers and fibrosis scores of hydronephrosis patients (*p ≤ 0.05 and **p ≤ 0.01).

A large fraction of obstructive nephropathies in children, with 50% of them being diagnosed before birth, represent pyelo-ureteral junction pathology. Kidney sections from surgical specimen from pediatric patients having undergone obstructive nephropathy surgery were analyzed for the inflammatory response by evaluating CD3^+^ T cell infiltration (Banff score) and fibrosis as well as MC infiltration using tryptase and chymase and c-kit as a marker. Three representative patients with different degrees of the inflammatory T cell infiltrate are presented in Figure 1A. A quantitative analysis of scores including 21 patients is shown in Figure 1B. The data indicate that patients, showing, respectively, a low, intermediate, and high inflammatory cell infiltrate (Banff scores 1, 2, and 3) are positively correlated with MC numbers. We also evaluated fibrosis scores using Masson’s Trichrome-stained sections. Data show no statistically significant fibrosis between the different Banff scores. Altogether, our data indicate that MCs infiltrate the kidney in UPJ patients and that their presence is correlated with the degree of inflammation, but not with the degree of fibrosis.

### Enhanced Renal Pathology in WT versus MC- and MCPT4-Deficient Mice after pUUO

**Figure 2:**
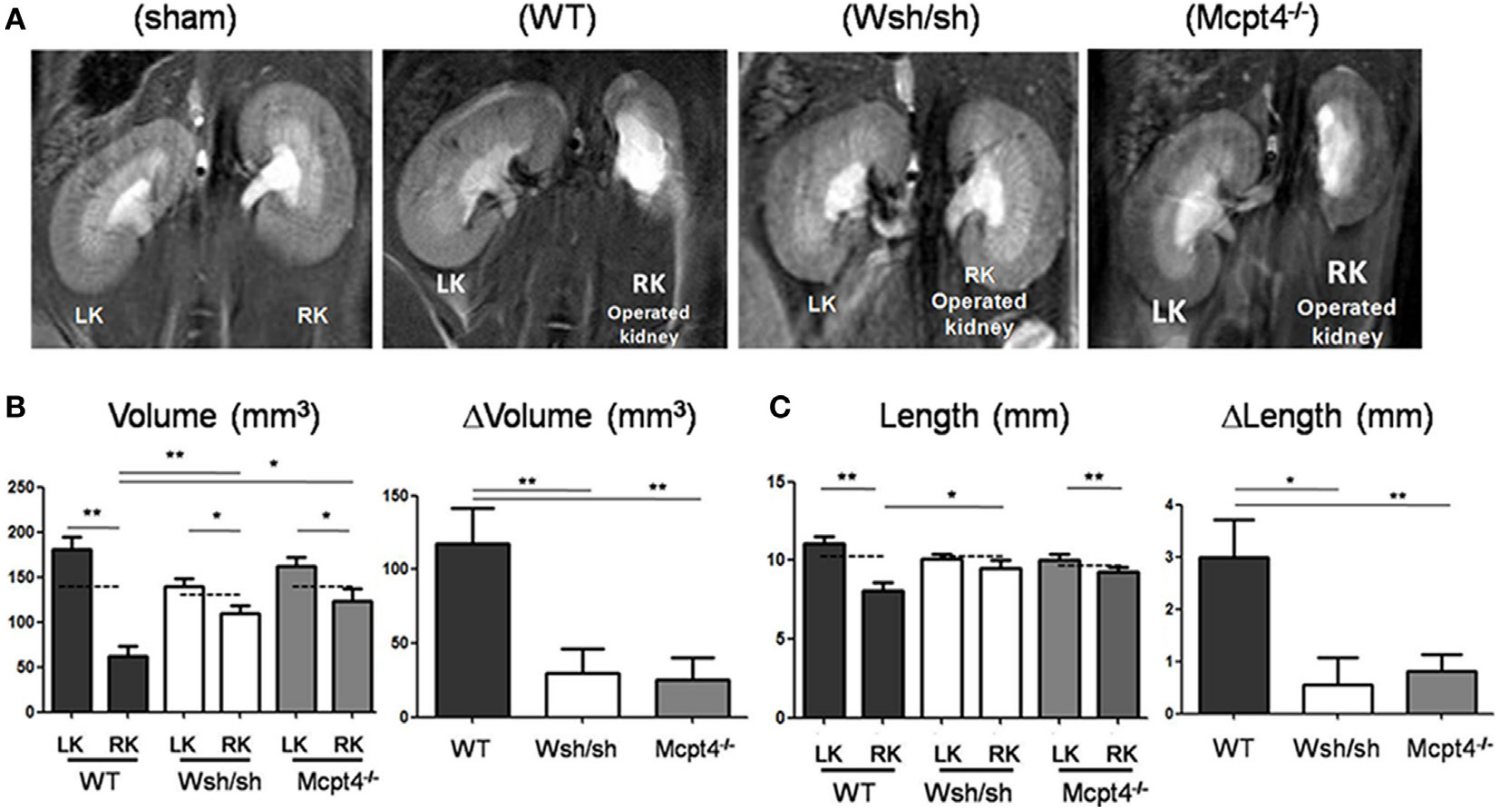
**Evaluation of renal pathology after partial unilateral ureteral obstruction (pUUO) by magnetic resonance imaging (MRI)**. **(A)** Renal MRI using T2-weighted coronal sequences for wild-type (WT), mast cell (MC)-deficient and MCPT4-deficient mice at D75 after surgery. LK = non-operated left kidney and RK = operated right kidney with pUUO. Note the differences in morphology between hypotrophic right kidneys (RKs) and hypertrophic left kidneys (LKs). **(B)** Quantification of volume for RK and LK of the indicated mice at D75 post-surgery (*n* = 8 for WT, 11 for MC, and 8 for MCPT4-deficient mice). The hatched line is the mean volume of control kidneys of from sham mice at D75. Delta volume (ΔV) denotes the difference in volume between hypertrophic LK and hypotrophic RK for each strain of mice. Data are the mean ± SEM of indicated numbers of mice [see **(A)**] (*p ≤ 0.05 and **p ≤ 0.01). **(C)** Quantification of length data for RK and LK of indicated mice at D75 post-surgery (*n* = 8 for WT, 9 for MC, and 12 for MCPT4-deficient mice). The hatched line is the length of control kidneys from sham mice at D75. Delta length (ΔL) denotes the difference in length between hypertrophic LK and hypotrophic RK. Data are the mean ± SEM of indicated numbers of mice [see **(A)**] (*p ≤ 0.05 and **p ≤ 0.01).

To analyze the role of MC in UPJ pathology, we used the previously developed pUUO model in neonatal mice (29, 30, 33). Following surgery at day 3 of life in WT, MC-, and MCPT4-deficient mice, a total of 21 WT, 15 W^sh/sh^, and 22 *Mcpt4^−/−^* mice survived. Out of these 17 WT, 12 W^sh/sh^ and 20 *Mcpt4^−/−^* mice presented pUUO as judged by ureter permeability test (see Materials and Methods) at the day of euthanization. To judge UPJ pathology progression, MRI sequences taken at day 75 after surgery the day before euthanization from WT, MC-, and MCPT4-deficient mice were analyzed. Representative morphologic T2-weighted images of left and right operated kidneys of the various mouse strains compared to sham-operated mice (WT) are presented in Figure 2A. In agreement with the partial obstruction UPJ pathology, WT mice developed marked differences in size between operated right kidneys (RKs) and non-operated left kidneys (LKs). Volume and length measurements of RK and LK in WT animals (Figures 2B,C) revealed a pronounced hypotrophy in operated RK as compared to kidneys from sham-operated mice. At the same time, a compensatory upregulation of length and volume occurred in the non-operated LK. These alterations were not seen in sham-operated animals as described before (30) (for quantitative data, see weight analysis below). Strikingly, our measurements showed that kidney hypotrophy and the compensatory hypertrophy were less pronounced in MC- and MCPT4-deficient strains. This became particularly evident when differences (Δ) between the LK and RK were plotted revealing significant differences between WT and MC-deficient or MCPT4-deficient strains.

**Figure 3:**
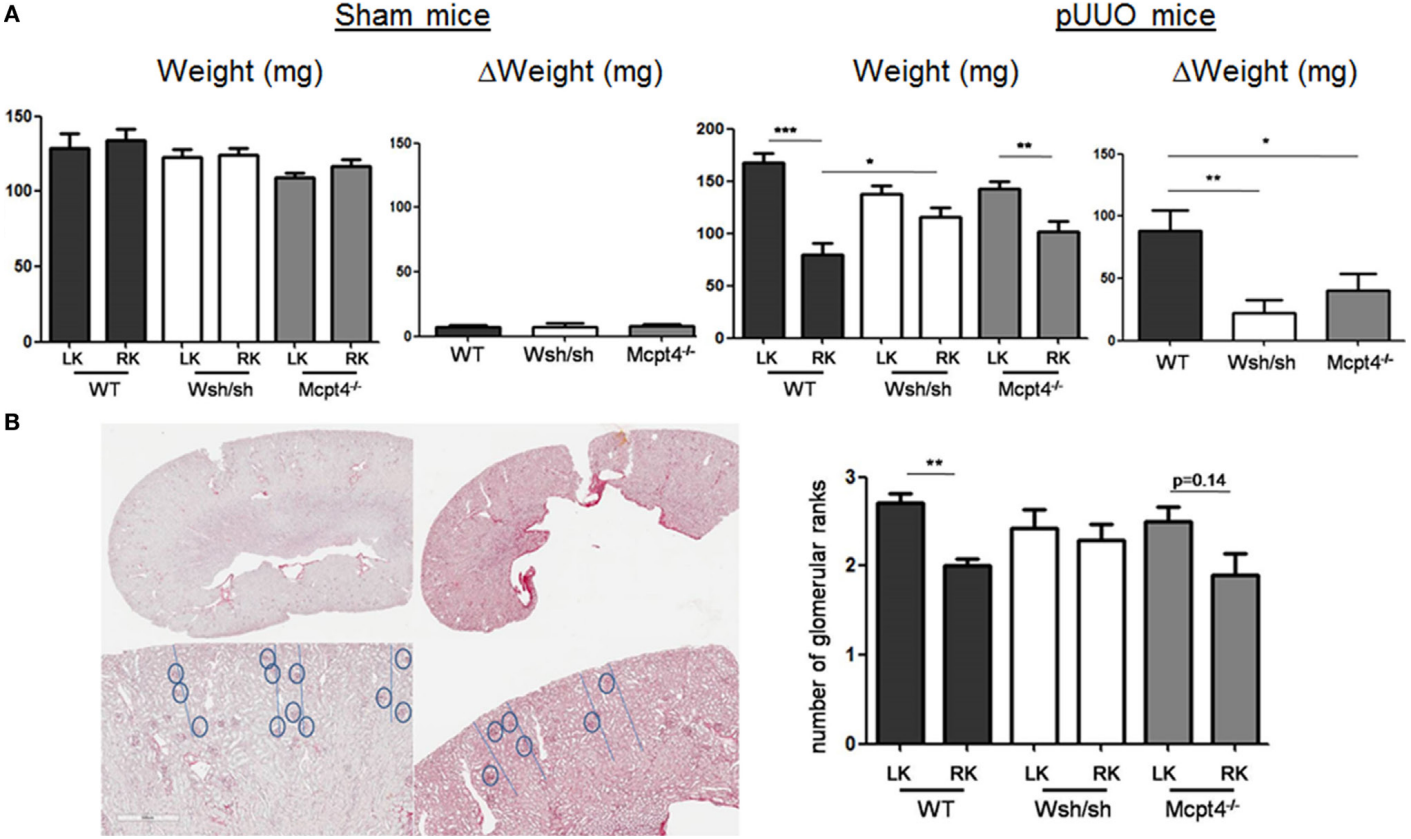
**Evaluation of renal pathology after partial unilateral ureteral obstruction (pUUO) by postmortem kidney weight and nephrogenesis analysis**. **(A)** Evaluation of kidney weight following pUUO. The left panel shows the quantification of weight for right kidneys (RKs) and left kidneys (LKs) of indicated sham-operated mice at D75 post-surgery [*n* = 4 for wild type (WT), 5 for mast cell (MC), and 5 for MCPT4-deficient mice]. The right panel shows the quantification of weight for RK and LK of indicated pUUO operated mice at D75 post-surgery (*n* = 17 for WT, 12 for MC, and 20 for MCPT4-deficient mice). Delta weight (ΔW) denotes the difference in weight between hypertrophic LK and hypotrophic RK for each mouse strain of mice. Data are the mean ± SEM of indicated numbers of mice (as above) (*p ≤ 0.05, **p ≤ 0.01, and ***p ≤ 0.001). **(B)** Left panel shows histological images of LK and RK (Sirius Red stain) from WT mice. The upper row shows the entire slice of kidney, the bottom row shows a magnified part of the cortex (×4) to illustrate the decrease in glomerular ranks for RK compared to LK (gray dashed line). The right panel shows the corresponding quantification of glomerular ranks in WT, MC-, and MCPT4-deficient mice. Data are the mean ± SEM of indicated numbers of mice [see **(A)**], **p ≤ 0.01.

To confirm development of kidney hypo- and hypertrophy, we determined kidney weights after euthanization. Surgery in general did not modify the normal growth of mice, as no significant differences in the average weight between each operated and sham-operated strain were found (WT = 24.93 ± 0.76 g versus 23.24 ± 1.43 g, W^sh/sh^ = 23.44 ± 0.73 g versus 23.08 ± 1.09 g, Mcpt4*^−/−^* = 22.73 ± 0.61 g versus 21.83 ± 0.49 g). By contrast, measurements of kidney weight confirmed the development of weight loss in operated RK and compensatory upregulation of weight in LKs versus those from sham-operated WT animals, while no differences were seen in sham-operated animals of all strains (Figure 3A). The weight loss and compensatory weight increases were diminished in MC- and MCPT4-deficient mice confirming that UPJ pathology was less severe in the absence of MC and MCPT4. For all strains, kidney weight significantly correlated with the kidney volume calculated on MRI volume sequences (Table 1). We also examined whether MC deficiency could influence normal kidney development after pUUO, based on the fact that, at the time of surgery, kidney nephrogenesis is incomplete, by determining the number of glomerular ranks. We observed a slight compression of the cortex in RK with diminution of the number of glomerular ranks, likely due to an arrest in nephrogenesis after pUUO (Figure 3B). This was particularly observed in WT mice, while no significant differences became apparent in MC and MCPT4-deficient strains supporting also a role of MC in nephrogenesis. Together, our data support that MC play a role in the development UPJ pathology including nephrogenesis.

**Table 1:** **Correlation coefficient analysis between kidney weights and kidney volumes of left kidneys (LKs) and right kidneys (RKs)**.

Mouse strain	*r*^2^ value LK	*r*^2^ value RK
Wild type	0.79*	0.95**
W^sh/sh^	0.93***	0.90**
*Mcpt4^−/−^*	0.81**	0.82**

*p ≤ 0.05, **p ≤ 0.01, ***p ≤ 0.001.

### MC- and MCPT4-Deficient Mice Show Decreased Fibrotic and Levels of Inflammatory Cellularity after pUUO

**Figure 4:**
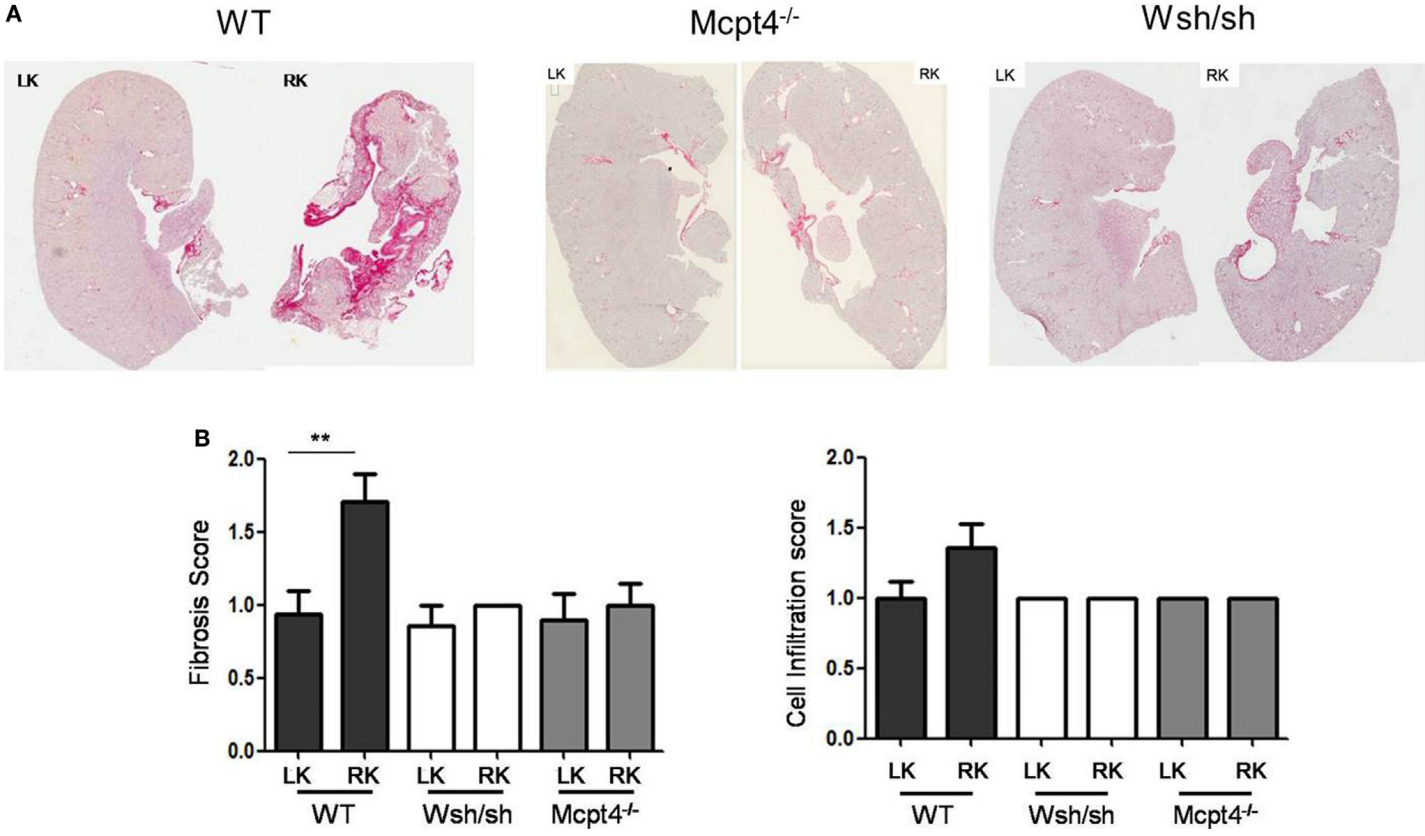
**Evaluation of fibrosis and cellular infiltration after partial unilateral ureteral obstruction**. **(A)** Representative images of Sirius Red-stained sections from operated right kidneys (RKs) and left kidneys (LKs) of WT, MC-deficient, and MCPT4-deficient mice at D75 post-surgery. **(B)** Quantitative analysis of fibrosis (left panel) and inflammatory cells infiltration (right panel) of LK and RK at D75 post-surgery. Data are the mean ± SEM of indicated numbers of mice [see panel **(A)**], **p ≤ 0.01.

**Figure 5:**
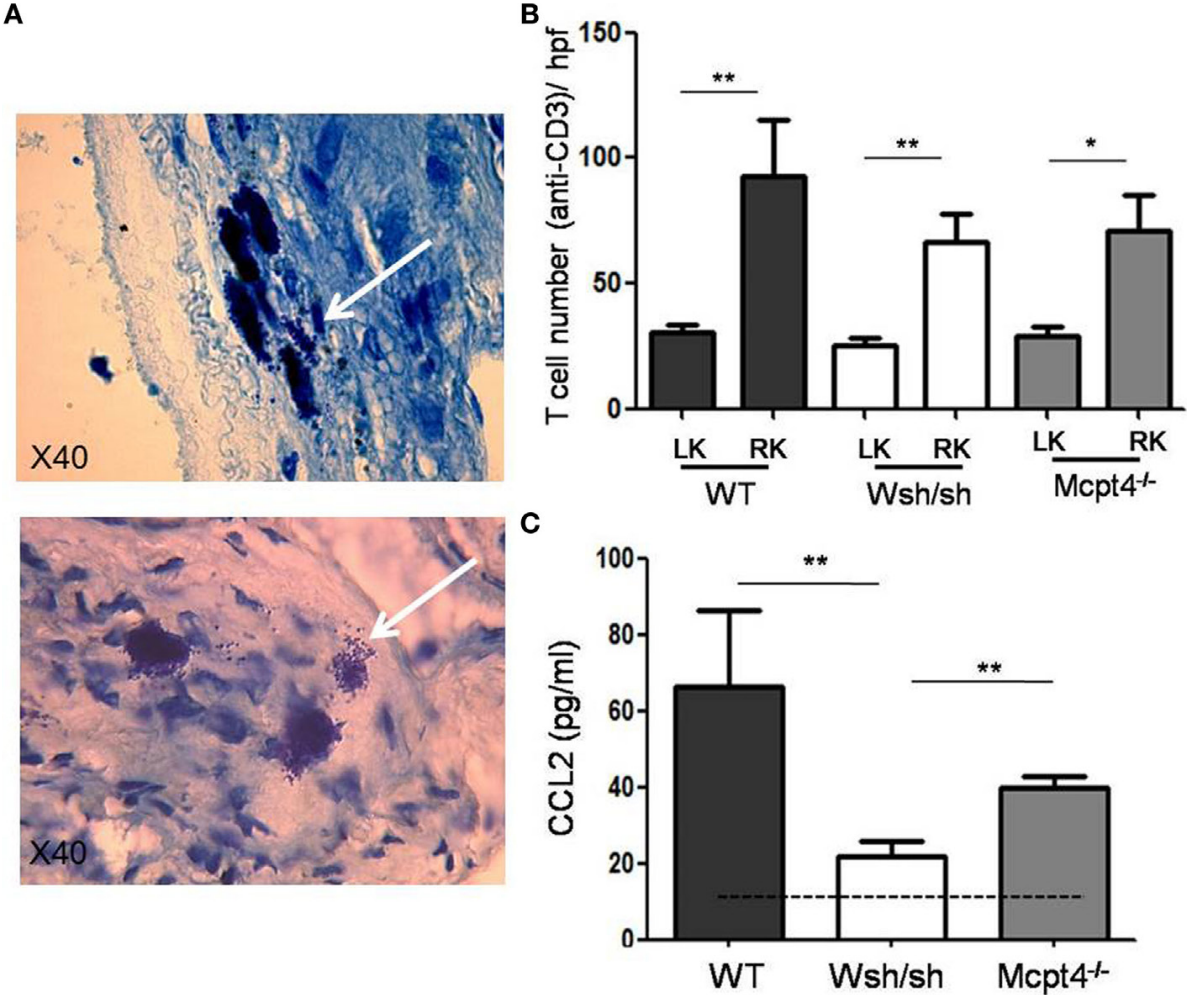
**Evaluation of the inflammatory response after partial unilateral ureteral obstruction**. **(A)** Representative photomicrographs of toluidine blue-stained sections of connective tissue of renal capsules obtained from right sham and right operated kidneys of wild-type (WT) mice D75 post-surgery. Note the presence of degranulated MCs in operated kidneys (arrow). **(B)** Quantification of T cell infiltrate (CD3 staining) in sham and operated right kidney parenchyma of WT (16), MC-deficient (11), and MCPT4-deficient (9) mice. Data are the mean ± SEM of indicated numbers of mice, **p ≤ 0.01 and *p ≤ 0.05. **(C)** Blood was drawn from sham (5), WT (6), MC-deficient (7), and MCPT4-deficient (11) mice at D75 post-surgery, and CCL2 levels were measured using an ELISA as described under Section “Materials and Methods.” Data are the mean ± SEM of indicated numbers of mice, **p ≤ 0.01. There is a significant difference between the three strains and sham mice (Kruskal–Wallis test, *p* = 0.0007).

Ureteropelvic junction pathology is characterized by the development of interstitial fibrosis, which is quite variable in patients and may develop over years being hardly to predict. In agreement, our histologic assessment of fibrosis (Figure 4A) after pUUO shows only discrete signs of fibrosis in WT mice appearing rather focal and localized mainly in the medulla in areas close to the pelvis dilations. Interestingly, when examining MC- and MCPT4-deficient strains, very little fibrosis and cell infiltration was apparent when compared to WT mice (Figures 4A,B). These data are in agreement with a fibrosis-promoting role of MC and MCPT4 chymase. Concerning the inflammatory cell infiltrate, MCs were not detectable in kidney parenchyma, but they could be detected in kidney capsules, where they often revealed a degranulated phenotype (Figure 5A). In kidney parenchyma, we evaluated T cell infiltration by staining sections with an anti-CD3 antibody. Although T cell infiltration was detectable after pUUO, their numbers did not differ between the various mouse strains (Figure 5B). No significant macrophage infiltration was found. Next, we evaluated systemic parameters of the associated inflammatory response by measuring serum CCL2 chemokine levels (Figure 5C) known to be increased in patients with UPJ (38, 39). Our results revealed increased levels of serum CCL2 levels in WT mice after pUUO when compared to sham control mice. The levels were also significantly elevated compared to MC- and MCPT4-deficient mice.

### MC- and MCPT4-Deficient Mice Show Decreased Epithelial–Mesenchymal Transition after pUUO

**Figure 6:**
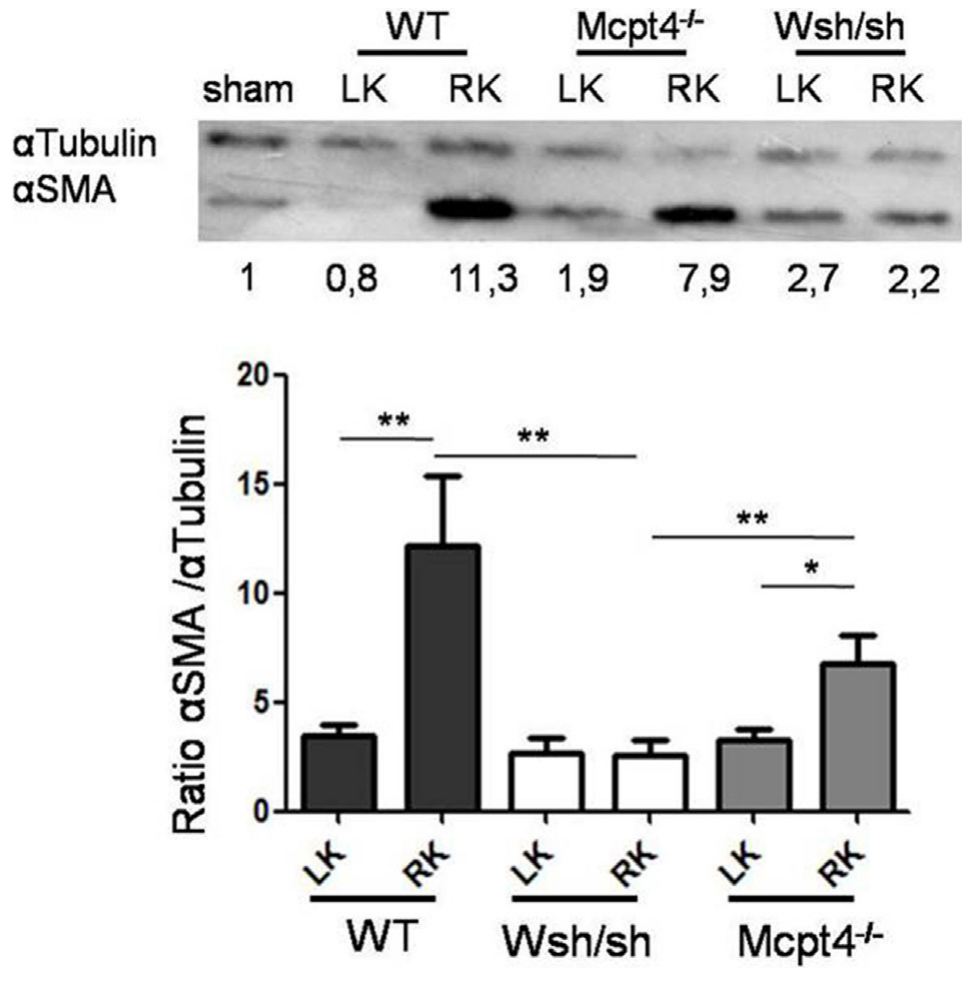
**Evaluation of α-SMA expression in kidney parenchyma after partial unilateral ureteral obstruction**. Upper panel shows representative western blot analysis of α-SMA expression in kidney parenchyma in WT (17), MC-deficient (10), and MCPT4-deficient (17) mice at D75 post-surgery. At D75 post-surgery, cell lysates were prepared from left and right kidneys and submitted to western blotting using an anti-α-SMA antibody. Anti-tubulin was used for analysis of equal loading. Numbers below the blot indicate ratio of α-SMA versus α-tubulin as compared to a sham kidney mouse used as a universal control in each gel and arbitrarily set to 1. Lower panel shows quantitative analysis of western blots. Data are the mean ± SEM of the indicated numbers of mice, **p ≤ 0.01.

As fibrosis development was rather focalized and moderate after pUUO, we concentrated on epithelial–mesenchymal transition (EMT) as an early step that precedes fibrosis development to further examine the MC contribution in pathology. EMT is characterized by the generation of myofibroblasts producing ECM proteins and α-SMA, which is abundantly expressed as shown by western blot analysis (Figure 6). Our quantitative analysis of operated RKs as compared to a sham control kidney loaded each time in parallel shows that consistent with EMT after pUUO WT mice show a marked increase in α-SMA levels. In comparison, MC-deficient mice show significantly less α-SMA, while MCPT4-deficient animals show intermediate levels. These results supported a role of MC in the early stages of myofibroblast generation, which is partially dependent on MCPT4.

**Figure 7:**
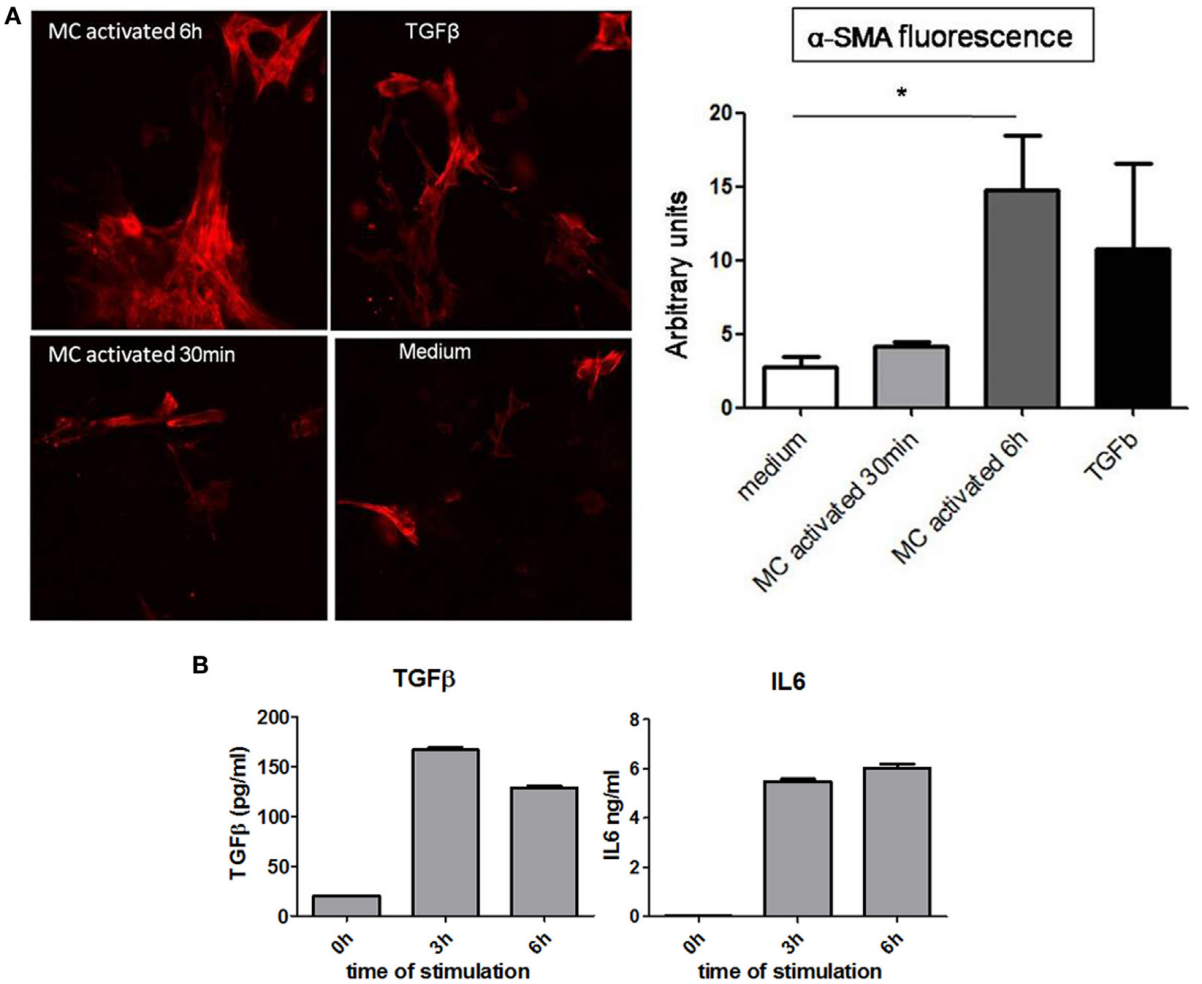
**Analysis of role of mast cell (MC) in inducing EMT in proximal kidney tubular cells**. **(A)** Representative images of α-SMA immunofluorescence and relative quantification using ImageJ software in kidney proximal tubular cells after (time of) incubation with medium alone, TGFβ (50 ng/ml) and supernatants from activated MC (30 min and 6 h) through the IgE receptor. Data are the mean ± SEM, *p ≤ 0.05. **(B)** IgE anti-DNP sensitized bone marrow derived MCs were washed and stimulated with 30 ng/ml of DNP-HAS. Supernatants were collected at indicated time points and cytokine concentrations were determined by ELISA subtracting the values in medium alone. Data are mean ± SD of triplicate values.

To further analyze the possible implication of MC in EMT, we examined the effect of supernatants collected from primary resting or activated MC on the expression of α-SMA by cultured proximal tubular cells. Immunofluorescence analysis (Figure 7A) shows that similar to the incubation with TGFβ, used as a positive control, supernatants from long-term (6 h) cultured MC enhance the expression of α-SMA. Activation does not further increase the expression. Supernatants from short-term activated MC (30 min) do not show this enhancing effect. This supports that MC constitutively secrete a factor promoting EMT. Further analysis (Figure 7B) shows that cultured BMMC upon stimulation for 3 and 6 h *via* the IgE receptor can indeed produce cytokines such as TGFβ and IL6 known to be implicated in EMT (40, 41).

## Discussion

The current challenge in congenital hydronephrosis or UPJ pathology is the indication for surgery, which depends on the degree of damage and the evolution of disease. Although progress has been made, current non-invasive imaging methods even when performed regularly after birth provide poorly reliable information when compared to invasive histological analysis (3, 42). Moreover, the physiological mechanisms involved in chronic renal disease and development of fibrosis are still poorly understood. Based on this and previous data indicating that MC represent potentially important players in renal diseases (8, 11, 43, 44), we decided to analyze the involvement of MC and one of its specific mediator, MCPT4 chymase, using available deficient mice (17, 45) in a recently established model of UPJ obstruction (29, 30). Our results show that WT mice present a more severe pathology when compared to MC-deficient mice, while MCPT4-deficient mice show an intermediate phenotype of disease. These results add to a series of studies showing on the role of MC in fibrosis development. While some reported that MC promote fibrosis development (22, 44, 46, 47) as shown here, others suggested that MC or derived mediators can have a protective role (14, 19). These differences may be explained by the specific pathophysiological context with different types of injuries but also disease kinetics. In the long-term disease model as presented here, MCs may ultimately favor fibrosis progression.

In agreement with previous data (30, 48), morphological kidney analysis of disease progression in the three mice strains revealed differences in kidney weights, length, and volume at the day of euthanization indicative of hypotrophy and compensatory hypertrophy in RKs and LKs, respectively. No such differences were found in sham-operated mice as observed before (30). Importantly, the operated RK organ atrophy in WT mice was significantly higher compared to MC- and MCPT4-deficient strains supporting that both MC and MCPT4 contribute to disease aggravation. This was confirmed by kidney length and volume analysis calculated from MRI sequences. Thus, volume and length determination by MRI sequences represented a rather good evaluation for kidney atrophy (operated RK) and kidney hypertrophy (LK) for all three strains of mice used as also confirmed by our correlation coefficient analysis between weight and volume measurements (Table 1).

Interestingly, pUUO also induced an abnormal nephrogenesis as quantified by a decrease in the number of glomerular ranks. Such abnormalities of glomerular development and decrease in kidney weights have previously been described in newborn mice with partial and complete UUO (25, 26, 49–51). Similarly, Gasser et al. (52) demonstrated, in the human fetus, developmental defects leading to a decreased number of nephrons in chronic obstructive kidney diseases. Strikingly, after pUUO, the difference in glomerular ranks became apparent only in WT mice while MC- and MCPT4-deficient mice do not show significant differences. This supports a possible implication of MC and MCPT4 in nephrogenesis, likely by their capacity to influence the tissue remodeling process during development.

Our data indicated a compensatory hypertrophy of contralateral LKs after pUUO as compared to sham controls. Differences (deltas) in volume length and weight between LK and RK were particularly found in WT mice, while they were attenuated in both MC and MCPT4-deficient mice in agreement with protection in the absence of MC and MCPT4 chymase. A similar compensatory hypertrophy was initially described in nephrectomy and complete ureteral obstruction animal models (53). Likewise, previous results from our team showed that compensatory hypertrophy becomes apparent 10 days post-surgery, and the phenomena then further increased during following weeks after pUUO and then decreased with age (30). The compensatory hypertrophy was pronounced in agreement with data indicating that they are more important when the earlier obstruction occurs in life (54). Overall, these morphological results confirm the importance of organ atrophy measurements to interpret the severity of kidney impairment in clinical practice.

While the morphological assessment readily showed differences, histological lesions appeared more discrete in WT mice and were barely detectable in MC- and MCPT4-deficient mice even at day 75 after pUUO. This was likely due to the minor parenchymal alterations in kidneys. In particular, inflammatory cell infiltration and fibrosis were focal and located relatively close to the renal cavities. These observations differ from what has been described in complete UUO models where parenchyma shows massive and diffuse lesions in the cortex and fast development (starting at D3) of fibrosis (29, 51, 55). Nevertheless, they allowed to discern differences between WT and MC- and MCPT4-deficient mice concerning cellularity and fibrosis and glomerular rank number. For all these parameters, they appeared to be very mild in MC- and MCPT4-deficient mice supporting an important role of MC and the MCPT4 protease in pUUO pathology. Based on the relative low levels of fibrosis, we investigated αSMA expression, a marker epithelial–mesenchymal transition (EMT) (56) as an early step in fibrosis development coinciding with myofibroblast generation. Our results revealed a significant increase of αSMA in WT mice, intermediate levels in MCPT4-deficient mice, and little expression in MC-deficient mice. This suggests that analysis of αSMA expression may be more adequate for the detection of early changes in UPJ pathology than evaluation of fibrosis.

The analysis of α-SMA expression supported a role of MC in myofibroblast generation. Myofibroblasts represent important precursor cells in fibrosis development (57). They are induced during the inflammatory phase through the effect of various cytokines and growth factors such as TGFβ, CTGF, IL6, and other factors. We, therefore, examined directly in co-culture experiments whether MCs were able to support myofibroblast generation. Our results showed that, like TGFβ, supernatants from cultured MC for 6 h and MC activated *via* IgE receptors supported the generation of myofibroblasts. By contrast, supernatants from short-term (30 min) activated MC did not support expression of αSMA. This could be in agreement with a minor and probably indirect role of MCPT4 in the epithelial–mesenchymal transition, as this protease is exclusively secreted from granular stores. We have also analyzed possible cytokines involved in this transition such as TGFβ (41) and IL6 (40). We found that stimulation of cultured MCs *via* the IgE receptor can induce the production of these cytokines in agreement with previously published data (47, 58).

Concerning the characterization of the inflammatory infiltrate, we focused on MCs as well as T cells in view of the massive filtration observed in patients. MCs were absent in mouse kidney parenchyma as already noted before in several types of kidney diseases (8, 11, 43) were present in kidney capsules of WT mice, where they often showed a degranulated phenotype, likely due to the pressure exerted by the accumulation of urine in the pelvis (59). Concerning T cells, albeit they were elevated, we did not see differences between the various strains of mice supporting that MCs were not majorly contributing to the differences in pathology. For the systemic inflammatory response, we found elevated levels of CCL2 in the serum of WT mice when compared to MC-deficient mice with MCPT4-deficient mice showing an intermediate phenotype. These data are in agreement with data in humans as it was shown that CCL2 was elevated in kidneys and urine of UPJO patients and support that MCs may contribute either directly or indirectly *via* MCPT4 (38).

In conclusion, our data on the pUUO mice model support a role of MC in the development of UPJ pathology based on studies in a relevant animal model. At least part of the action may be due to the chymase MCPT4 as mice deficient in this protease show an intermediate phenotype when compared to MC-deficient mice. One major activity by MC in the development of UPJ pathology may include their capacity to induce myofibroblast generation during the early stages of fibrosis development. During developmental stages, as the case for human pathology, MC and MCPT4 may also impact pathology by their capacity to enhance tissue remodeling. Our data further supported that MRI imaging allowed a good evaluation of kidney impairment in particular when combined with examination of both kidneys. They also support that histological assessment should include early markers of fibrosis development as fibrotic lesions are often focal and may not necessarily be included in biopsy specimen.

## Ethics Statement

All experiments were performed in accordance with the national ethical guidelines and with the approval of local authorities of the Comité d’Éthique Expérimentation Animale Bichat-Debré. All patients have provided informed consent for surgery and follow-up histological analysis according to the institutional regulations.

## Author Contributions

MP, LA, WB, LD, LM, SV and JC performed experiments; MP and MA performed and analyzed IRM; JC and MP performed and analyzed histology; MP, LA, M-LP-M, AEG set up surgery model for pUUO; MÅ and GP provided mouse strain and advise; AEG and UB supervised the work; MP, M-LP-M, AEG, and UB wrote the manuscript.

## Conflict of Interest Statement

The authors declare that the research was conducted in the absence of any commercial or financial relationships that could be construed as a potential conflict of interest. The reviewer, KM, declared a shared affiliation, though no other collaboration, with the authors MP, LA, WB, LD, LCM, JC, UB to the handling editor, who ensured that the process nevertheless met the standards of a fair and objective review.
